# Active Paper Sheets Including Nanoencapsulated Essential Oils: A Green Packaging Technique to Control Ethylene Production and Maintain Quality in Fresh Horticultural Products—A Case Study on Flat Peaches

**DOI:** 10.3390/foods9121904

**Published:** 2020-12-19

**Authors:** Antonio López-Gómez, Alejandra Navarro-Martínez, Ginés Benito Martínez-Hernández

**Affiliations:** 1 Food Safety and Refrigeration Engineering Group, Department of Agricultural Engineering, Universidad Politécnica de Cartagena, Paseo Alfonso XIII 48, 30203 Cartagena, Murcia, Spain; alejandra.navarro@upct.es; 2 Biotechnological Processes Technology and Engineering Lab, Campus Muralla del Mar, Instituto de Biotecnología Vegetal, Universidad Politécnica de Cartagena, Edif I+D+I, 30202 Cartagena, Murcia, Spain

**Keywords:** β−cyclodextrin, inclusion complex, carvacrol, respiration, quality, phenolic compounds

## Abstract

Plant essential oils (EOs) have several bioactive properties, highlighting their high antimicrobial and antioxidant capacities. As such, the use of EOs in active packaging has received special attention in the last few years. Nevertheless, the inhibitory effect of EOs on quality-degrading enzymatic systems of plant products during postharvest life has not been deeply studied. The effects of an EO active paper sheet on ethylene biosynthesis and quality (and related quality-degrading enzymes) of flat peach (*Prunus persica* var. platycarpa) samples were studied during 5 days (continental terrestrial transport) or 26 days (long maritime transport) storage at 2 or 8 °C, both followed by commercialization simulations (4 days at 22 °C). EOs released from active packaging reduced ethylene production by 40–50%, and by up to 70% after commercialization periods. These results were correlated with lower 1-aminocyclopropanecarboxylic acid (ACC) content and ACC-oxidase activity. Physicochemical fruit quality (as indicated by soluble solids content, titratable acidity, color, and firmness) was also better preserved by EO active sheets due to enzymatic inhibition (polygalacturonase and polyphenoloxidase). Furthermore, phenolic compounds (mainly catechin and cyanidin-3 glucoside) and total antioxidant capacity were increased (by up to 30 and 70%, respectively) in EO-packaged samples after 8 °C storage and the subsequent commercialization period. Conclusively, EO active paper sheets controlled ethylene production in flat peaches, maintained fruit quality, and even increased health-promoting bioactive compounds.

## 1. Introduction

The flat peach (*Prunus persica* var. platycarpa) is a mutation from the standard peach, which is characterized by its oblate shape and fewer skin trichomes, with reddish-pink skin and a white stem area on the fruit’s shoulders (close to the peduncle area). Flat peaches have received several names, such as the *paraguayo*, *chato*, doughnut peach, and Saturn peach, among other names. Flat peaches are very similar to platerines (a mutation of nectarines), but the latter ones are characterized by the absence of skin trichomes. Flat peach flesh is characterized by its hard and *crocant* texture, similar to nectarines. Nevertheless, textural changes due to enzymatic activity (e.g., polygalacturonase (PG) and pectinmethylesterase) occur during the postharvest life of flat peaches, as generally observed in stone fruits, leading to flesh-softening disorders such as mealiness, which occur during cold storage of stone fruits [1]. Flat peach flesh is also characterized by its white color. However, flesh color disorders may occur in flat peaches, such as enzymatic browning (oxidation of phenolic compounds, mainly by polyphenoloxidase (PPO) activity) and flesh reddening (mainly due to the accumulation of flavonoids) [2,3]. Thus, fruit industry losses due to fruit browning are estimated at 50% [3]. Flat peach sweetness and acidity are also considered as important quality parameters for this stone fruit, together with flesh texture and color [4,5]. In addition to the organoleptic characteristics, the high content of antioxidant compounds (mainly phenolic compounds) in flat peaches has also contributed to the popularity of this stone fruit of Asian origin [5].

Stone fruits ripen and deteriorate quickly at ambient temperature, with cold storage being the main postharvest technique conventionally used to extend their shelf life. Thus, cold storage close to 0 °C is recommended, although the “killing temperature zone” (2.2 and 7.5 °C) of stone fruit may be avoided to prevent accelerated metabolism [1]. However, the combination of cold storage with other postharvest techniques may extend the product shelf life, with increasing interests in and need for sustainable treatments of natural origin that are free of chemical substances.

Plant essential oils (EOs) have been conventionally considered as excellent postharvest treatments due to their high antimicrobial properties. Carvacrol is the major component of oregano EOs, with wide activity against both Gram-negative (e.g., enterobacteria) and Gram-positive bacteria, as well as other microbial groups such as molds [6,7,8,9]. Interestingly, essential oil (EO) mixes, including the major EO components (e.g., carvacrol), together with their correspondent EO (e.g., oregano EOs), have shown a synergistic effect in terms of their antimicrobial activity [10]. EOs are considered as strong antioxidants, which are also able to inhibit the activity of quality-degrading enzymes such as PPO [11], although such inhibitory effects of EOs on quality-degrading enzymes have not been deeply studied. Furthermore, several EO components were able to inhibit ethylene (widely known as the “ripening hormone”) production in peach seed tissue [12]. In particular, one study [12] also found that the content of 1-aminocyclopropanecarboxylic acid (ACC), the substrate of the ACC oxidase (ACO) involved in ethylene biosynthesis, was also reduced with EOs. Nevertheless, no further studies were conducted to deeply study the effects of EOs during postharvest storage of horticultural products on ethylene production, ACC content, and ACO activity, the key rate-limiting enzyme in the ethylene biosynthesis pathway, as recently reviewed [13]. Unfortunately, EOs have characteristic strong flavors, low water solubility, and high volatility, which have greatly limited their application at the industrial scale [14,15]. However, nanotechnology has emerged in the postharvest area in recent years, with a special focus on the encapsulation of EOs to reduce their oxidation and evaporation, while ensuring controlled EO release in small concentrations to the surrounding atmosphere of fruits and vegetables. Active packaging is an emerging technology that extends the product shelf life through controlled release of an encapsulated compound [16]. Thus, active EO packaging with eco-sustainable materials (e.g., paper and cardboard) can extend the shelf life of several fruits and vegetables [7,17,18,19]. Furthermore, high relative humidity (RH), such as that maintained in cold rooms used for fruits and vegetables, has been reported to increase the controlled EO release from the EO–β-cyclodextrin (EO−*β*CD) inclusion complex [18,20].

This work aimed to study the effects of an active EO (encapsulated within β-cyclodextrin) paper sheet on flat peach quality during cold storage and after a commercialization simulation (room temperature). Furthermore, the effects of released EOs from the active material were firstly studied in detail on quality-degrading enzymes and key substrates and enzymes of the ethylene biosynthesis pathway. The responses to EOs of the main health-promoting (bioactive) compounds were also studied.

The hypothesis of this study was that the controlled EO release from an active packaging material would reduce ethylene production in plant products based on the inhibition of related enzymes and substrates involved in the ethylene biosynthesis pathway. Such inhibitory effect of EOs could also affect other enzymes, such as those related to product quality degradation (e.g., PPO and PG), meaning the product shelf life will consequently be extended.

## 2. Materials and Methods

### 2.1. Materials

Flat peach (*Prunus persica* var. platycarpa, cv. Filoe) samples were obtained from the company Agronativa S.L. (Cieza, Murcia, Spain) in July 2020. Flat peaches were grown in open fields under organic agriculture practices. The fruit were manually harvested and transported to the pilot plant of the Agricultural Engineering Department of the Universidad Politécnica de Cartagena, where they were selected (according to the company recommendations for this flat peach cv.) with homogeneous size (82–86 mm diameter), weight (160–190 g), color (yellowish areas less than 30% of fruit surface), physical integrity, and absence of decay. Selected fruit were stored at 2 or 8 °C overnight in open plastic boxes to ensure the stabilization of fruit metabolism, and the experiment was conducted the next day.

An EO mix composed of carvacrol/oregano EO/cinnamon EO 70:10:20 (weight (*w*):*w*:*w*) (EO composition analysis previously reported [21]) (Lluch Essence S.L.; Barcelona, Spain) was prepared based on the high antimicrobial effect of these EOs, in accordance with our previous studies [7,22]. The EO−*β*CD inclusion complex was prepared using the kneading method [23]. Briefly, 0.15 g of EOs was mixed with 1.14 g of *β*CD (1:1 molar ratio) (Kleptose^®^10 Roquette; Lestrem, France) in a mortar with 3 mL of ethanol, then kneaded for 45 min, and finally maintained in a vacuum desiccator at room temperature for at least 72 h. This EO−βCD inclusion complex has been fully characterized by a high encapsulation efficiency ranging from 92 to 95% [7,21].

Active paper sheets containing the former EO−βCD inclusion complex were prepared at an industrial scale by the company Papeles El Carmen S.A.U. (Navarra, Spain). Recycled Kraft paper (weight 80 g m^−2^) was used. The EO−βCD inclusion complex was dissolved in water prior to spraying on paper. Water containing the EO−βCD inclusion complex was then sprayed to obtain 1 g m^−2^ of the EO−βCD inclusion complex on these active paper sheets. No EO-related off-flavors were transferred to fruit samples (based on preliminary informal sensory tests), as previously described in our publications with these concentrations of EOs within the active packaging of other stone fruits [19].

### 2.2. Experiment Description

An active EO paper sheet (38 × 15.5 cm) was introduced in a glass jar (Mason-type; 2 L capacity), whereby the internal perimeter of the glass jar was covered with the active sheet side (side sprayed with the EO−βCD inclusion complex). Then, four fruit samples (169 ± 17 g per fruit) were introduced in the glass jar containing the paper sheet. Control treatment (CTRL) lacked the active EO sheet. Then, the four fruit samples were stored at 2 or 8 °C, leaving the jar lid half-closed to avoid fruit dehydration and O_2_ reduction–CO_2_ accumulation (O_2_ ≥ 18% and CO_2_ < 1%; measured with a portable CO_2_/O_2_ analyzer (Checkpoint; PBI Dansensor, Ringsted, Danemark)) for 5 and 26 days. The cold storage period of 5 days was selected to simulate a short continental terrestrial transport, while the cold storage period of 26 days was selected to simulate long storage and overseas transport [17,24]. A commercialization simulation (COM; 4 days at 22 °C) was also conducted after each cold storage period to observe possible physiological disorders due to cold storage and real conditions during the commercial fruit retail period [1,2,24]. Three jars (three replicates) were prepared per treatment (active EO sheets or CTRL), storage temperature (2 or 8 °C), and sampling time (5 days, 5 days + COM, 26 days, 26 days + COM) (192 fruit samples + 20 fruit samples (initial analyses)).

At each sampling time, ethylene production, respiration (expressed as CO_2_ production), physicochemical determination (soluble solid content (SSC), pH, and titratable acidity (TA)), skin color, and flesh firmness were measured. Furthermore, samples were fast-frozen (liquid N_2_) and stored at −80 °C until analysis of ACC content, enzymatic activities (ACO, PG, and PPO), phenolic compounds, and total antioxidant capacity.

### 2.3. Ethylene Production and Respiration

The ethylene and CO_2_ production from flat peach samples was determined following the procedure described previously [25]. Briefly, glass jars containing the samples (4 fruit samples) were closed with the hermetic lids (which already contained a sampling port for gas analysis) and ethylene and CO_2_ were allowed to accumulate for 2 and 1 h, respectively. Gas sampling (1 mL) was done from the headspace of jars using a gas-tight syringe, then the gases were injected into a gas chromatograph instrument (GC; Clarus 500 GC; PerkinElmer Inc., Shelton CT, USA) for ethylene and CO_2_ analysis_._ Two measurements (technical replicates) were made per jar.

The GC instrument contained a column system for ethylene determination (stainless steel column packed with Porapak Q (1/8″, 80/100 mesh size; Teknokroma, Barcelona, Spain)) and a 2-column system for CO_2_–O_2_ determination (a stainless steel column packed with HayesepQ (1/8″, 80/100 mesh size; Teknokroma) followed by a stainless steel column packed with a 5A molecular sieve (1/8″, 80/100 mesh size; Teknokroma)), both connected to independent sampling ports. The GC conditions for ethylene were: oven, injector, and flame ionization detector temperatures of 80, 120, and 250 °C, respectively; with synthetic air and H_2_ as gas carriers at 350 and 35 mL min^−1^, respectively. The GC conditions for CO_2_ determination were as follows: oven, injector, and thermal conductivity detector temperatures of 80, 120, and 200 °C, respectively; with synthetic air and H_2_ as gas carriers at 30 and 2 mL min^−1^, respectively. Gas calibration was done by comparison with an external ethylene–CO_2_ mixture (gas molar fraction 10 ppm/10%) standard (Praxair, Molina de Segura, Spain). Ethylene and CO_2_ production were expressed as nmol kg^−1^ h^−1^ and nmol kg^−1^ s^−1^, respectively.

### 2.4. Physicochemical Analyses and Weight Loss

Juice from flat peaches (pulp + skin) was obtained with a blender (model MX2050; Braun, Marktheidenfeld, Germany), which was then filtered with a 4-layer cheesecloth. SSC was determined with a digital handheld refractometer (model N1; Atago, Tokyo, Japan) at 20 °C and expressed as °Brix. A pH meter (Basic20; Crison, Alella, Spain) was used to measure the pH of the fruit juice. The TA of the diluted juice (10 mL plus 40 mL of distilled water) was determined with an automatic titrator (model T50; Metter Toledo, Milan, Italy) with 0.1 M NaOH to reach pH 8.1. TA was expressed as malic acid in g kg^−1^.

Color was determined on fruit skin using a colorimeter (Chroma Meter CR−400, Konica Minolta, Tokyo, Japan) at illuminant D65 and a 2° observer, and with a viewing aperture of 8 mm. Three measurements were made for each fruit sample, which were automatically averaged by the device. The following specific color index (Equation (1) [26]) was selected as the most appropriate for this flat peach cv. of reddish-pink coloration.

(1) $$\mathrm{Color}\;\mathrm{index}=\frac{180-{}^{\circ}\mathrm{Hue}}{L^{*}+\mathrm{Chroma}}$$

where L*, °Hue, and Chroma are the luminosity, hue angle and chroma index, which were calculated from a* and b* CIE color parameters [26].

Pulp firmness was determined with a Texture Analyzer instrument (TA XT Plus; TA Instruments, Surrey, UK) by measuring the amount of force (expressed in N) needed to penetrate fruit flesh (after skin removal) 15–17 mm deep with a probe of 8 mm Ø. For this, flat peaches were previously cut (perpendicular to the equatorial diameter) in halves and placed (without stone) on the Texture Analyzer base. Three fruit samples were analyzed per each jar, with one measurement performed for each fruit half.

The weights of fruit samples were monitored at each sampling time to determine the weight loss (%) of fruit samples during storage periods.

Fruit samples were regularly examined to detect rotten samples and were considered as infected if a visible lesion was observed. Rotten samples were then discarded and not included for the rest of the analyses. Decay incidence was expressed as a percentage of fruit units infected related to the initial total number of fruit units.

### 2.5. 1-Aminocyclopropane-1-Carboxylic Acid (ACC)

Free ACC and N-malonyl-ACC (MACC) contents were analyzed according to the original method of Lizada and Yang [27], as reviewed by Bulens et al. [28]. For ACC extraction, 4 g of ground (mill IKA A11 Basic; IKA, Königswinter, Germany) frozen tissue (with liquid N_2_) was homogenized (T-18 digital ULTRA-TURRAX^®^, IKA, Königswinter, Germany) with salicylic acid (4% in distilled water), then vortexed and left on ice for 30 min in agitation. Afterwards, samples were centrifuged (3090× *g*, 10 min, 4 °C) and used as the ACC extract (free ACC). MACC content was obtained through the difference of total ACC (after acid hydrolysis of samples) minus free ACC. For acid hydrolysis, 0.2 mL of 6 M HCl was added to 0.5 mL of ACC extract, vortexed, and then left for 3 h in a water bath at 99 °C. Samples were cooled down at room temperature and 0.2 mL of 6 M NaOH was then added. Finally, samples were centrifuged (22,000× *g* for 5 min) and the obtained supernatant was used as the hydrolyzed ACC extract (total ACC).

For ACC quantification, 1.4 mL of free ACC extract (0.1 mL in the case of hydrolyzed ACC extract) was mixed with 0.4 mL (0.2 mL for the hydrolyzed ACC extract) of 10 mM HgCl_2_ in a 20 mL glass GC vial, then quickly sealed with an encapsulable septum. Then, reaction started after addition (with a syringe) of 0.2 mL (0.1 mL for the hydrolyzed ACC extract) of a NaOCl (5% *volume* (*v*):*v*)/NaOH (6 M) mix (2:1 *v*:*v*) and followed by incubation for 4 min in ice. Finally, 1 mL from the GC vial headspace was injected in the GC instrument and the produced ethylene quantity was obtained (described in Section 2.3). To obtain the stability efficiency of ACC during acid hydrolysis, duplicate samples were doped with 10 µL of 50 µM ACC (commercial standard). ACC (free and MACC) contents were expressed as nmol g^−1^.

### 2.6. Enzymatic Analyses

#### 2.6.1. ACC Oxidase (ACO)

ACO activity was analyzed as previously described [28,29]. For ACO extraction, 0.5 g of ground frozen sample (plus 50 mg of polyvinylpolypyrrolidone) was added to 1 mL of 100 mM Tris-HCl Buffer (including 10% glycerol (*w:v*) and 30 mM sodium ascorbate; pH 7.2) and agitated for 15 min at 4 °C. Samples were then centrifuged (22,000× *g,* 30 min, 4 °C) and the supernatant was used as the ACO extract. ACO reaction was initiated by mixing 0.4 mL of the ACO extract with 3.6 mL of ACO reaction buffer (12.5 mM Tris-HCl buffer containing 10% glycerol, 1 mM ACC standard, 10 mM sodium ascorbate, 50 µM iron sulphate, 10 mM sodium bicarbonate, and 1 mM dithiothreitol; pH 7.2) in a 20 mL glass GC vial, which was then quickly sealed with an encapsulable septum. Reaction continued for 1 h at 30 °C (water bath). Finally, 1 mL from that headspace was injected in the GC instrument and the produced ethylene was determined. ACO activity was expressed as ethylene produced in nmol g^−1^ h^−1^.

#### 2.6.2. Endo-Polygalacturonase (endo-PG) and Exo-Polygalacturonase (exo-PG)

Endo-PG and exo-PG activities were analyzed based on the method used by Pressey and Avants [30], with some modifications. For enzyme extraction, 2.5 g of ground frozen sample (plus 75 mg of polyvinylpolypyrrolidone) was added to 7.5 mL of 0.5 M NaCl extraction buffer and homogenized (Ultraturrax; low speed) for 10 s at 2 °C. Samples were then centrifuged (14,000× *g,* 30 min, 4 °C) and the supernatant was used as the enzyme extract. First, 20 µL of 0.2 M acetate buffer (pH 4 for endo-PG and pH 5.4 for exo-PG), 20 µL of enzyme extract, and 40 µL of polygalacturonic acid (washed with ethanol) solution (prepared in 0.2 M acetate buffer (pH 4 or 5.4)) were placed in a flat-bottom 96-well microplate (UV-STAR, Greiner Bio-One, Frickenhausen, Germany). Additionally, 20 µL of 4 mM CaCl_2_ was added for the exo-PG reaction. The reaction was continued for 1 h at 37 °C in a microplate reader (Infinite M200; Tecan, Männedorf, Switzerland). Subsequently, the reaction was stopped by addition of 200 µL of 100 mM borate buffer (pH 9). Reducing groups formed during the reaction were quantified by cyanoacetamide reaction [31] by adding 40 µL of 1% 2-cyanoacetamide solution, covering the plate with an adhesive film (Q-StickTM pPCR Seal; 4titude, Surrey, UK) to avoid evaporation during subsequent heating, then placing the covered microplate within a hermetic plastic bag. Cyanoacetamide reaction was conducted for 10 min at 100 °C in a water bath. Cyanoacetamide reaction was immediately stopped by immersion in ice water for 30 s. Finally, absorbance was measured at 276 nm in the microplate reader. A standard curve with D-galacturonic acid was prepared (0.01–2.45 mg mL^−1^), which was assayed by being placed 20 µL of such standard solution with 60 µL of distilled water, 200 µL of 100 mM borate buffer (pH 9), and 20 µL of 1% 2-cyanoacetamide solution following the reaction (100 °C for 10 min), as previously described. One endo-PG or exo-PG activity unit (U) is defined as the amount that catalyzes the formation of 1 µmol of reducing groups in 1 h at 37 °C.

#### 2.6.3. Polyphenoloxidase (PPO)

PPO activity was analyzed based on previous literature [3,32], with some modifications. For enzyme extraction, 2.5 g of ground frozen sample (plus 75 mg of polyvinylpolypyrrolidone) was added to 7.5 mL of 100 mM sodium acetate (including 1% Triton X-100 and 1 M NaCl; pH 7) and homogenized (Ultraturrax; low speed) for 10 s at 2 °C. Samples were then centrifuged (14,000× *g,* 30 min, 4 °C) and the supernatant was used as the enzyme extract. First, 235 µL of 50 mM sodium acetate (pH 7), 35 µL of enzyme extract, and 235 µL of reaction buffer (freshly prepared before the assay) (5 mM l-3,4-dihydroxyphenylalanine (L-DOPA), 0.065 mM ethylenediaminetetraacetic acid, and 2.1 mM ascorbic acid (sodium salt) in 1:1:1 (*v:v:v*)) were placed in a flat-bottom 96-well microplate (UV-STAR, Greiner Bio-One, Frickenhausen, Germany). PPO reaction (at 30 °C) was monitored for 10 min (every 1 min) in the microplate reader at 265 nm. PPO activity was calculated as the slope in the linear part of the obtained activity curve. One PPO activity unit (U) is defined as the decrease in absorbance of 0.001 units at 265 nm.

### 2.7. Bioactive Compounds

A single extract was prepared for total phenolic content, individual phenolic compounds, and total antioxidant capacity. For this, 2.5 g of frozen ground sample was homogenized (Ultraturrax; 10 s) in 7.5 mL of 80% MeOH (including 2 mM NaF). Extraction was followed by incubation (1 h, 5 °C) on an orbital shaker (Stuart SSL1; Stuart, Osa, UK) at 60 cycles per min^–1^. Samples were then centrifuged (14,000× *g*, 10 min, 4 °C) and supernatants were used as phenolic compounds or TAC extracts.

#### 2.7.1. Phenolic Compounds

The Folin–Ciocalteu reagent method was used to analyze the total phenolic content, as previously described [33]. Briefly, 19 μL of the previous extract was placed on a flat-bottom polystyrene (PS) 96-well plate and 29 μL of 1 N Folin–Ciocalteu reagent was added. The obtained mixture was incubated for 3 min at room temperature in darkness. After incubation, 192 μL of a solution containing 38 mM Na_2_CO_3_ and 500 mM NaOH was added and the reaction was carried out for 1 h at room temperature in darkness. Then, absorbance was measured at 750 nm using the microplate reader. Total phenolic content was expressed as gallic acid equivalents (GAE) in g kg^–1^ (fresh weight basis). Each of the three replicates was analyzed in triplicate.

The individual phenolic compounds of the extracts were identified and quantified as previously described [34]. An ultra-high-performance liquid chromatography (UHPLC) instrument (Shimadzu; Kyoto, Japan) was used, which was equipped with a DGU–20A degasser, LC–170 30AD quaternary pump, SIL–30AC autosampler, CTO–10AS column heater, and SPDM– 20A photodiode array detector (DAD). Chromatographic separation was carried out using a Gemini C18 column (250 mm × 4.6 mm, 5 μm particle size; Phenomenex, Macclesfield, UK). Four mobile phases (A: water:MeOH, 95:5, v:v; B: water:MeOH, 88:12; C water:MeOH, 20:80; D: 100% MeOH) were used, as previously described [35]. All mobile phases were acidified (5%) with formic acid. Elution started with 100% A, which remained isocratic until minute 5. The elution gradient started with 100% A; 0–10 min: 100% B linear; isocratic for 3 min; 13–35 min: 75% B and 25% C linear; 35–50 min: 50% B and 50% C linear; 50–52 min: 100% C linear; isocratic for 5 min; 50–52 min: 100% C linear; then the column was washed with 100% D at 60 min. The flow rate was 1 mL min^–1^. UV–visible detection was performed at 510 and 340 nm for anthocyanins and hydroxycinnamic acid derivatives, respectively. Identification of phenolic compounds was conducted by UHPLC–triple quadrupole electrospray mass spectrometer (MS) (6420 Waters Acquity Triple Quad, Waters Corp., Milford, CT, USA) and compared to previously published MS data and UV spectra. Nitrogen was used as a nebulizing gas at 50 psi (345 kPa) and 300 °C [35]. The column and chromatographic conditions were the same as those used for the UHPLC–DAD analysis. Commercial standards (Merck, Darmstadt, Germany) were used to prepare the external standard curves. Individual phenolic compounds were expressed in mg kg^–1^ (fresh weight basis). Each of the three replicates was analyzed in duplicate.

#### 2.7.2. Total Antioxidant Capacity

Total antioxidant capacity was determined using the free radical 2.2-Diphenyl-l-pict3,1hydrazyl (DPPH^●^) [36]. Total antioxidant capacity determination of extracts was conducted as previously described [33]. Briefly, a solution of 0.7 mM DPPH was prepared in methanol for 2 h before the assay and adjusted to 1.10 ± 0.02 nm immediately before use. A 21 µL aliquot of the extract was placed on a flat-bottom PS 96-well plate and 194 µL of the adjusted DPPH solution was added. The reaction was carried out for 30 min at room temperature in darkness and the absorbance at 515 nm was measured using the microplate reader. Total antioxidant capacity was expressed as equivalents of 6-hydroxy-2,5,7,8-tetramethylchroman-2-carboxylic acid (Trolox) in µmol/kg (fresh weight basis). Each of the three replicates was analyzed in duplicate.

### 2.8. Statistical Analyses

The data were subjected to analysis of variance (ANOVA) using SPSS software (v.19 IBM, New York, NY, USA). Statistical significance was assessed at *p* = 0.05, and Tukey’s multiple range test was used to separate the means.

## 3. Results

### 3.1. Ethylene Biosynthesis

**Figure 1:**
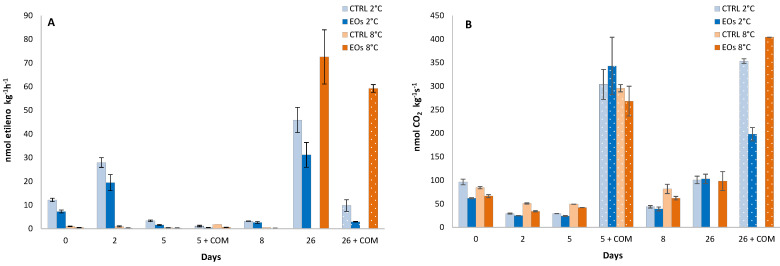
Ethylene production (**A**) and CO_2_ production (**B**) during cold storage periods (2 and 8 °C) and complimentary commercialization simulations (COM; 4 days at 22 °C) for flat peach samples with or without active paper sheets (including βcyclodextrin (CD)-encapsulated essential oils (EOs)) (*n* = 3 ± SD).

Initial ethylene production values for flat peaches were 12.2 and 1.0 nmol kg^−1^ h^−1^ at 2 °C and 8 °C, respectively (Figure 1A), following previous literature on flat peaches [4,37]. The higher ethylene production trend at 2 °C compared with 8 °C may be explained as a physiological response of flat peaches to low storage temperatures. Thus, previous studies with other fruits and vegetables have found that low storage temperatures may act as an abiotic stress, with similar metabolic responses as in our data [38,39,40]. In addition, the higher ethylene production at 2 °C compared to 8 °C could be also explained by the proximity to the “killing temperature zone” for stone fruit, which ranges between 2.2 and 7.5 °C, and is characterized by accelerated stone fruit metabolism [1]. This abiotic stress related to low refrigeration temperatures has been previously correlated with increased CO_2_ production (respiration rate) at early stages (<48 h of storage) at lower temperatures, which changes to the opposite behavior (higher respiration rate as the refrigerated temperature increases) [38,39]. Thus, the storage temperature×time interaction was significant for CO_2_ (and ethylene) production, showing the same trend—initial (<48 h) higher respiration at lower storage temperature and then higher respiration rate at a higher temperature during the rest of the storage period (Figure 1B).

The storage time factor and the storage temperature×storage time interaction were significant for ethylene and CO_2_ production (Table 1). As such, ethylene production remained low from day 2 until day 26, after which an increment was observed with 46 nmol kg^−1^ h^−1^ in CTRL samples stored at 2 °C for 26 days. Such behavior is characteristic of climacteric fruits, such as peaches and other stone fruits [2,4,37,41]. No ethylene or CO_2_ production was measured in CTRL samples stored at 8 °C for ≥26 days due to the incipient physiological disorders observed (see Section 3.4), which led to altered ethylene and CO_2_ production results with high variability. The subsequent commercialization period for samples previously stored for 5 days did not show remarkable ethylene differences (<2 nmol kg^−1^ h^−1^). Nevertheless, maximum CO_2_ production was observed after the commercialization period of sample storage for 5 days, with levels of 270–340 nmol kg^−1^ h^−1^. A similar CO_2_ production peak was observed after the commercialization of samples stored for 26 days. The earlier CO_2_ production peak compared with the ethylene peak was in accordance with the initial increased CO_2_ production at the beginning of storage (<48 h). Fernández-Trujillo et al. [2] also reported more pronounced CO_2_ production compared with ethylene production in flat peaches stored for 6 days at 2 °C followed by a commercialization period. Those authors hypothesized that this low ethylene response was probably owing to damage in the ethylene synthesis system at low storage temperatures.

Packaging treatment and packaging treatment×storage temperature interaction were significant for ethylene production (Table 1), with samples stored with active EO sheets showing lower values than CTRL samples (Figure 1). In particular, samples stored with active EO sheets exhibited up to 32% and 70% lower ethylene production than samples with CTRL sheets after 26 days at 2 °C and after the corresponding commercialization period, respectively. The beneficial effect of EOs released from the active sheets were not able to be observed due to the the low ethylene production of samples at 8 °C after >26 days. Nevertheless, the supposed high ethylene production of CTRL samples stored for ≥26 days might suggest an inhibitory effect of released EOs against ethylene production in flat peaches. It has been demonstrated that EOs inhibited ethylene biosynthesis in several fruits and vegetables, although the mechanism behind this was not deeply studied regarding the key components (ACC, MACC, and ACO) of the ethylene biosynthesis pathway [12,42,43,44]. A similar trend was observed for CO_2_ production, probably owing to downregulated metabolism due to EO-induced ethylene inhibition. This hypothesis is reinforced according to the key components of the ethylene biosynthesis pathway—ACC and ACO, as explained in the following lines.

**Figure 2:**
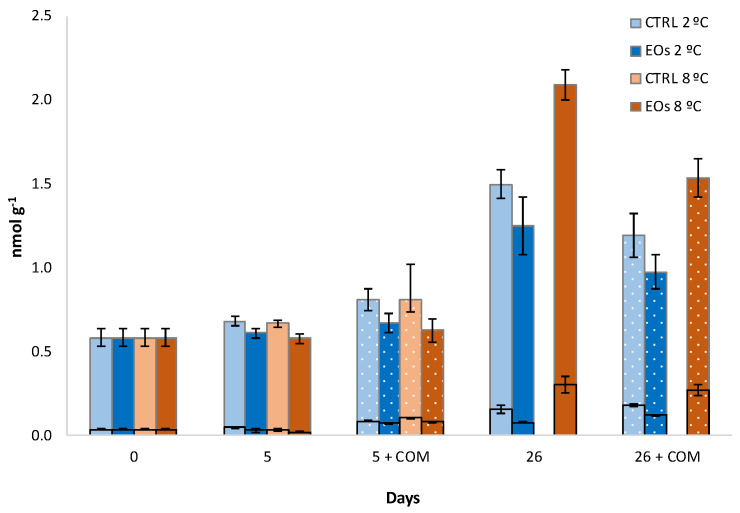
MACC and free ACC (black squared bars) contents during cold storage periods (2 and 8 °C) and complimentary commercialization simulations (COM; 4 days at 22 °C) for flat peaches with or without active paper sheets (including βCD-encapsulated EOs) (*n* = 3 ± SD).

The total ACC (free ACC + MACC) and MACC contents of 0.61 and 0.58 nmol g^−1^, respectively, on processing day were assessed (Figure 2). MACC represented 80–95% of the total ACC content, which is in accordance with the experiments of Hoffman and Yang [45], who discovered in 1982 that ACC is not only transformed into ethylene, but that a great amount of the formed ACC is converted into MACC. Similarly, Pretel et al. [37] observed that MACC accounted for >80% of total ACC content. MACC and ACC contents followed the same behavior as explained for ethylene production during storage of flat peaches. The ACC content of flat peaches, and stone fruit in general, is low in the preclimacteric stage and increases concurrently with the increase in ethylene production, indicating that the rate of ACC synthesis increases, surpassing the rate of ACC utilization for ethylene biosynthesis [37,46]. The high ACC accumulation in ripening fruits may be explained by the location of ACO in the plant cells; it is supposed that ACO is associated with the membrane, while the ACC-forming enzyme (ACC synthase) is located in the cytosol, although such hypotheses still remain a matter of debate, as recently reviewed by Houben and Van [13]. In this sense, ACC accumulation in ripening fruits could be due to the inefficiency of ACC transport between the site of formation and the site of utilization, as previously hypothesized by Brecht and Kader [46]. The hereby observed lower ethylene production (Figure 1) due to the released EOs from active sheets is in accordance with MACC and ACC contents, with the packaging treatment factor (and its interaction with storage time) being significant for both ACC and MACC (Table 1). Thus, the MACC content was 84% lower in fruits stored with active sheets after 26 days at 2 °C compared with CTRL samples. Although ethylene production was highly reduced after the commercialization period following the cold storage of 26 days, MACC and ACC contents did not show such high changes. This finding, which was also observed by Pretel et al. [37], may be explained by the accumulated ACC in plant cells, as previously explained. Thus, the hypothesis of ethylene inhibition by EOs may involve the key enzyme of the ethylene biosynthesis pathway—ACO, which is responsible of ACC conversion into ethylene.

**Figure 3:**
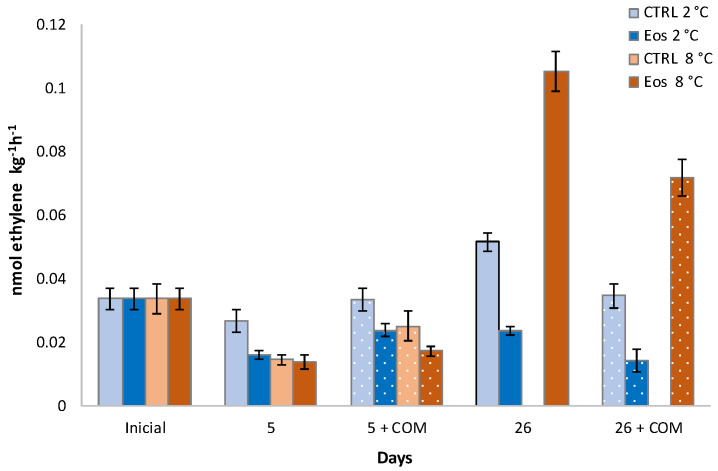
ACO activity during cold storage periods (2 and 8 °C) and complimentary commercialization simulations (COM; 4 days at 22 °C) of flat peaches with or without active paper sheets (including βCD-encapsulated EOs) (*n* = 3 ± SD).

Samples showed an initial ACO level of 0.037 nmol ethylene g^−1^ h^−1^ (Figure 3), which is in accordance with the initial ACO levels of other stone fruits such as nectarines [46]. Storage temperature, packaging treatment, and storage time, as well as all their interactions, were significant for ACO data (Table 1). ACO activity was high at day 26, followed by a decrease during its corresponding commercialization period, even at such high temperatures where enzymatic activities are enhanced. A similar postclimacteric diminution of such enzymatic activity has been described in nectarines and other climacteric fruits [46,47]. Similarly, Wu et al. [48] found that ACO gene expression of peaches exhibited maximum activity after 10 days at 4 °C followed by a decrease to initial levels, which were maintained for the rest of storage (20 days). Our data show that ACO activity was inhibited even at the early storage stage, with 41% lower ACO activity in samples packaged with active EO sheets compared to CTRL samples after 5 days at 2 °C. This inhibitory effect on ACO activity was even increased to 54% after 26 days at 2 °C. Furthermore, the ACO activity rates of samples with active EO sheets were 30 and 60% lower compared to CTRL samples after commercialization periods, which had previously been cold-stored for 5 and 26 days respectively. An inhibitory effect of EOs on browning-related enzymes (PPO, peroxidase, and phenylalanine ammonia-lyase) has already demonstrated, showing competitive inhibition of EOs within the active sites of phenylalanine ammonia–lyase in lettuce [11,49]. Therefore, the inhibitory effects of EOs on ethylene production could also be partially be due to competitive inhibition of the released EOs between active sheets and the active sites of other key enzymes of the ethylene biosynthesis pathway. In this sense, further experiments are needed to study such competitive inhibition of EOs between the active sites of ACO and other enzymes (e.g., ACS) of the ethylene biosynthesis pathway.

### 3.2. Soluble Solids, pH, and Titratable Acidity

Flat peaches showed initial SSC, pH, and TA values of 12.0 °Brix, 4.2, and 4.2 g L^−1^, respectively, on processing day (Table 2), which are in accordance with previous data on flat peaches [4]. Neither storage temperature, packaging treatment and storage time factors, nor their interactions were significant for SSC data (Table 1), with an SSC range of 12.0–12.7 °Brix. Nevertheless, an increasing SSC trend was observed throughout storage. The storage time factor was significant for pH and TA, which increased and decreased, respectively, during storage. Nevertheless, pH and TA changes were lower than 0.8 and 0.2 units, respectively, after 26 days of cold storage; and lower than 0.16 and 0.4 units, respectively, after the commercialization periods. Organic acids are metabolized during the postharvest life of fruits as an energy pool for respiration processes or are converted to sugars and carbon skeletons for the synthesis of new compounds, leading to the observed pH–TA increment–reduction.

Sugars, which are commonly responsible for sweetness in fruits, did not correlate well with sweetness in nectarines and peaches, however the sugar/organic acid (i.e., SSC/TA) ratio did [50,51]. In this sense, the SSC/TA ratio is highly recommended as a maturity index for stone fruit (and a wide range of horticultural products), with SSC/TA values at harvest for *paraguayo* of 3.1–3.5 [4], which are in agreement with our data (Table 2). SSC/TA differences among CTRL and samples with active EO sheets were lower than 0.5 SSC/TA units after 5 days of cold storage and the corresponding commercialization period. Nevertheless, EO samples showed 1–1.2 lower SSC/TA units than CTRL samples after 26 days, and 0.7 lower SSC/TA units after the corresponding commercialization period. Such beneficial effects of released EOs from the active sheets may be due to the lower respiration rates in these samples, leading to lower organic acids consumption as an energy pool.

### 3.3. Color, Appearance, and Decay

**Figure 4:**
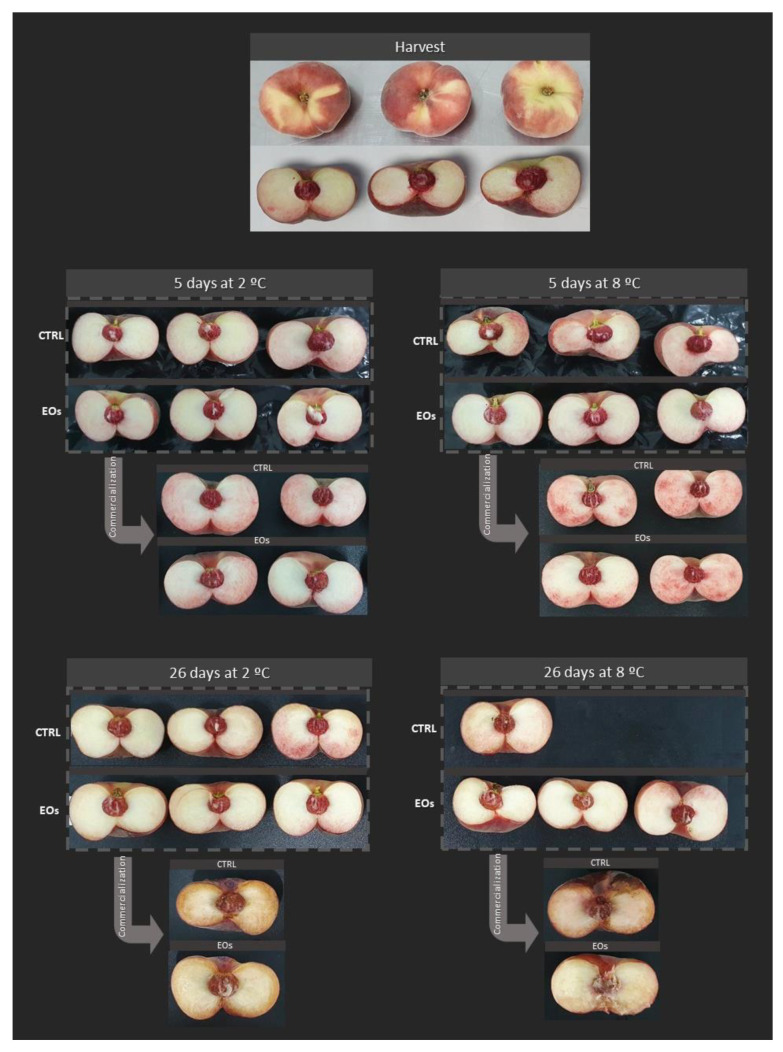
Flat peaches during cold storage periods (2 and 8 °C) and complimentary commercialization simulations (COM; 4 days at 22 °C) without (CTRL) or with active paper sheets (EOs) (*n* = 3 ± SD).

Color is a maturity index that is highly appreciated in stone fruit, with particular relevance to flat peaches, since the new varieties have higher reddish-pink skin coloration compared to previous varieties, which tended to be more whitish. Such reddish-pink skin coloration turns to darker tonalities throughout postharvest life. Thus, the color index “(180 − °Hue) × (L* + Chroma)”, developed for red table grapes [26], was used for flat peach skin color, since Carreño et al. [26] found that that color index was highly correlated (*R*^2^ = 0.98) with yellow > pink > red > violet > dark violet fruit changes. Flat peach skin showed an initial color index of 2.1 (Table 2), which corresponds to light pink-red tonality. Packaging treatment factor and the packaging treatment×storage time interaction were significant for color index data (Table 1). Samples packaged with active EO sheets did not show significant color index changes through storage periods, contrary to the color of CTRL samples, which turned into red-violet tonalities with increased color index values of 2.7–2.9 after 26 days (Table 2). Nevertheless, *paraguayo* skin appearance is very heterogeneous, with whitish-yellowish areas (see external appearance of fruits at harvest in Figure 4), which makes the study of the *paraguayo* flesh color more interesting.

Flat peach flesh may show color alterations related to physiological disorders at cold storage temperatures, such as flesh browning or flesh reddening. Flesh browning is due to oxidation of phenolic compounds by PPO enzymes, while flesh reddening in flat peaches may be linked to tissue senescence (leading to tissue disruption close to the skin and anthocyanin migration to the flesh) or a phenolic (anthocyanins) biosynthesis response to abiotic stresses, such as long, cold storage periods [1,52,53]. No visual flesh browning or remarkable flesh reddening was observed after 5 days of storage (Figure 4). However, mild flesh browning (in mesocarp regions close to the skin) was observed after 26 days, especially in samples stored at 8 °C, with lower incidence in samples with active EO sheets (Figure 4). Flesh browning in samples stored for 26 days was more evident after the subsequent commercialization period, with lower incidence in EOs samples at 2 °C, while CTRL and EOs samples stored at 8 °C showed very poor flesh quality with evident mealiness. Flesh reddening was observed after commercialization of samples cold-stored for 5 days, which might be explained by anthocyanin accumulation (see Section 3.6), as previously reported [52]. Nevertheless, such flesh reddening was not observed after the commercialization of samples stored for 26 days, probably owing to phenolic oxidation due to higher PPO activity (see Section 3.6).

Decay incidence was higher than 10% after 26 days. Specifically, CTRL samples stored at 2 °C showed a decay incidence of 25%, while samples packaged with active EO sheets showed only 15% decay after 26 days. The same trend was observed after commercialization of samples stored for 26 days at 2 °C, with lower incidence observed with released EO treatment. Such beneficial effects of EOs against decay were even observed after 26 days at 8 °C, with a decay incidence of 30%. The decay incidence increased to 70% in CTRL samples. Nevertheless, the commercialization of samples stored for 26 days at 8 °C induced decay symptoms in all samples (either CTRL or EOs). 

The antimicrobial effect against fruit decay of released EOs from inclusion complexes included in active packages has been previously observed by our research group for several horticultural products (tomatoes, peppers, lettuce, grapes, mandarins, nectarines, etc.), with decay incidence reductions of 1.3–1.5-fold after 25 days at 2 °C [19]. Interestingly, this effect could be explained, since ethylene is known to promote fungal growth, as observed for *Botrytis cinerea* in apples [54]. Finally, the antimicrobial effect of encapsulated EOs combined with cyclodextrins in active packaging has been widely studied [55,56,57].

### 3.4. Weight Loss

The weight loss in samples was ≤0.5% during the first 5 days of cold storage (Table 2). Storage temperature, packaging treatment, and storage time factors, as well as all their interactions, were significant for weight loss data (Table 1). Commercialization of samples cold-stored for 5 days led to weight losses of 2.6–2.7% in CTRL samples, while weight losses of samples packaged with active EO sheets were lower than 1.1%. Further cold storage led to weight loss of 5.5% after 26 days at 8 °C in CTRL samples, while the weight loss of CTRL samples after 26 days at 2 °C was 4%. As observed, low storage temperature in cold rooms, together with high RH, plays a crucial role in slowing down the physiological processes responsible for product weight loss (mainly dehydration) in horticultural products [58,59,60]. Shrivelling of flat peaches and flat nectarines is considered when weight losses of 3–5% are reached [4]. Nevertheless, visual shrivelling of CTRL samples was only appreciated (but to a low degree of intensity) after the commercialization period for samples stored for 26 days at 2 and 8 °C, with weight losses of 6.5 and 10% being observed, respectively. Weight loss was reduced by the EOs released from active sheets during cold storage (<1.3% after 26 days at 2 or 8 °C) and the corresponding commercialization periods compared to CTRL samples. Weight loss was still controlled by EOs after the commercialization period for samples stored for 26 days at 2 and 8 °C, with 4.7 and 4.8 less weight loss units, respectively, compared with CTRL samples.

We recently found that EOs (carvacrol, oregano EO, and cinnamon EO; encapsulated with a similar inclusion complex with β-cyclodextrin) released from active cardboard boxes reduced the weight loss of nectarines during cold storage [19]. Likewise, Santoro et al. [61] found that thyme and savory EO vapor treatments on peaches and nectarines reduced fruit weight loss during cold storage. Montero-Prado et al. [57] also found that cinnamon EOs (non-encapsulated) included in active plastic containers seemed to reduce weight loss rates in peaches during cold storage. Similar weight loss inhibition with EOs (carvacrol, eugenol, thymol, menthol, eucalyptol, oregano EO, and cinnamon EO) has been found for other horticultural products [17,18,19,44,62]. Although the mechanism of this protective effect of EOs against weight losses in horticultural products is still not well known, Montero-Prado et al. [57] found that lipoxygenase activity was inhibited in peaches stored within active EO (non-encapsulated) plastic containers. It is well known that lipoxygenase activity causes the peroxidation of the lipids contained in the cellular membrane, thus affecting the integrity (firmness) and resulting in mechanical and chemical changes (e.g., membrane ion leakage and membrane lipid content), as previously observed in peaches and peppers [63,64]. Hence, the beneficial effect of EOs on weight loss control may be found from the inhibitory effect of EOs on membrane-degrading enzymes, with PG (whose response to EOs has not been previously studied) playing a crucial role in stone fruits such as peaches, as will be elucidated in Section 3.5.

### 3.5. Firmness and Polygalacturonase Activity

An initial fruit firmness value of 25.9 N was found (Table 2), which was close to the range reported for ripened flat peaches [2,4]. Storage temperature, packaging treatment, and time factors, as well as temperature×time and packaging treatment×time interactions, were significant for firmness data (Table 1). In accordance, fruit firmness decreased during storage with values lower than 7 N, classifying this flat peach variety as a “melting” stone fruit according to Lurie and Crisosto (“melting fruit will soften to below 8 N firmness, while non-melting fleshed fruit will soften to 16 N or higher”) [1]. The firmness reduction was increased during fruit storage at 8 °C (Table 2), with evident mealiness after commercialization (Figure 3). Effectively, Lurie and Crisosto [1] stated that mealiness develops not in cold storage, but during the subsequent ripening period at warm temperatures. Mealiness in peaches has been characterized as thin cell walls similar to juicy fruit and large intercellular spaces filled with gel [65]. 

Samples packaged with active EO sheets showed better firmness retention during cold storage—and even after commercialization periods—with 1.1–1.3-fold higher firmness compared to CTRL samples. In accordance, we previously found that tomato firmness was better maintained during storage when using active cardboard boxes including the same EO–*β*CD inclusion complex [21,66]. The flesh firmness of stone fruit, unlike other fruits, is not consistently related to the full development of the organoleptic characteristics, but it is a quality index that highly influences the consumer purchase. In particular, stone fruit firmness is reduced during storage periods due to changes in the cell wall and middle lamella, and is also correlated with the activity of cell-wall-degrading enzymes such as PG [52].

**Figure 5:**
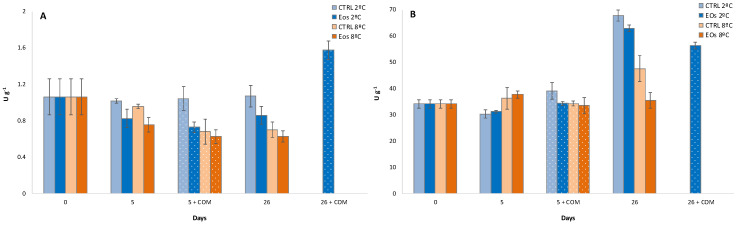
Endo-PG (**A**) and exo-PG (**B**) activities during cold storage periods (2 and 8 °C) and complimentary commercialization simulations (COM; 4 days at 22 °C) for flat peaches with or without active paper sheets (including βCD-encapsulated EOs) (*n* = 3 ± SD).

Initial endo-PG and exo-PG activities of 1.1 and 34.1 U g^−1^, respectively, were found (Figure 5). Pressey and Avants [30] earlier reported that clingstone peach varieties, such as flat peaches, contain high levels of exo-PG and very low endo-PG levels, with high retention of protopectin. Indeed, endo-PG attacks the linear polygalacturonase chains of proptopectin randomly and effectively reduces its molecular size, leading to the weakening of the cell walls and separation of cells observed in freestone peaches [30]. Higher exo-PG activity than endo-PG was also previously observed in flat peaches [2].

The storage time factor and the storage temperature×time interaction were significant for endo-PG activity (Table 1). Hence, endo-PG was reduced during storage of flat peaches (Figure 5), as has been widely reported in the literature (reviewed by Lurie and Crisosto [1]). Nevertheless, a sharp endo-PG increase was observed after the commercialization of samples stored for 26 days, in agreement with the observed mealiness in those samples (see Section 3.3). Furthermore, previous studies showed that mealiness did not develop during cold storage of stone fruit, but it did during the subsequent commercialization period at higher temperatures [1]. Endo-PG was lower during storage at 8 than 2 °C (Figure 5), which was not correlated with firmness data (Table 2). Similarly, endo-PG activity was not considered as a good index, since it was not closely linked with textural changes in previous studies with flat peaches [2,67]. Packaging treatment factor was also significant for endo-PG activity, but the packaging treatment×storage time interaction was not significant for endo-PG activity (Table 1). Thus, the released EOs from the active sheets led to an inhibitory effect of endo-PG activity compared to CTRL up to 30%. Competitive inhibition of EOs within the active sites of PPO in lettuce has already demonstrated [11]. A hypothetical inhibitory mode of action of EOs on PG active sites may be proposed, although it needs further investigation.

Similar to endo-PG, storage temperature, packaging treatment, and storage time factors were significant for exo-PG activity (Table 1). Storage temperature × time and packaging treatment × time interactions were also significant for exo-PG activity. Nevertheless, exo-PG did not show high activity changes (<7 U) after 5 days of storage or the subsequent commercialization period. Nevertheless, sharp increases of exo-PG activity of 99 and 40% were observed after 26 days of storage at 2 and 8 °C, respectively. The observed higher pectolytic activity at 2 °C was in accordance with the observed higher mealiness in these samples (as a consequence of the storage at low temperatures), as previously reported in the literature (reviewed by Lurie and Crisosto [1]). Similar to endo-PG, released EOs from the active sheets reduced exo-PG activity, showing 25% and 7% lower activity than CTRL samples after 26 days at 8 and 2 °C, respectively. The higher exo-PG inhibition during storage at 8 °C may be explained by a higher amount of EOs released from the inclusion complex at this higher temperature, as previously reported in the literature [18,20]. As discussed for endo-PG, a hypothetical inhibitory mode of action of EOs on PG active sites may occur, although further specific research is needed.

### 3.6. Polyphenoloxidase Activity and Phenolic Content

**Figure 6:**
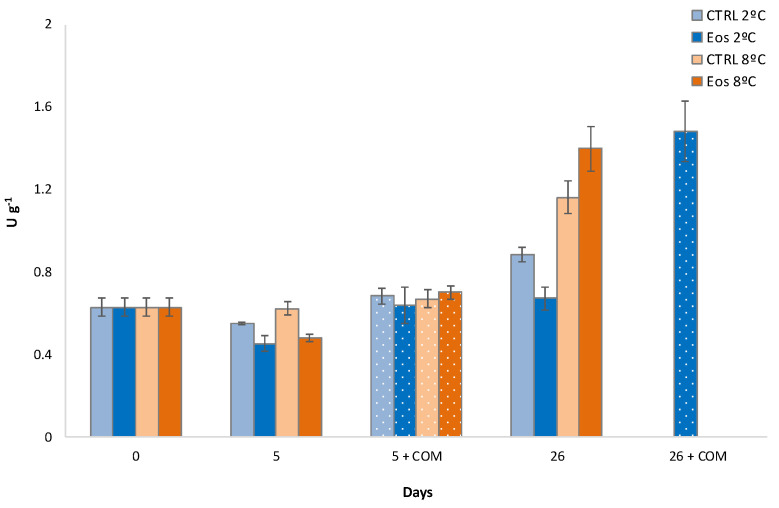
PPO activity during cold storage periods (2 and 8 °C) and complimentary commercialization simulations (COM; 4 days at 22 °C) for flat peaches with or without active paper sheets (including βCD-encapsulated EOs) (*n* = 3 ± SD).

PPO is a major enzyme present in horticultural products, which causes enzymatic browning with the oxidation of polyphenols into quinones. Flat peach samples showed an initial PPO activity of 0.63 U g^−1^ (Figure 6). Storage temperature and storage time factors were significant for PPO activity (Table 1). Furthermore, all factor interactions were significant—including the packaging treatment×storage time interaction—for PPO activity. In particular, the highest PPO increments were observed after 26 days at 8 and 2 °C with 85 and 40% higher PPO activity values in CTRL samples compared to EO samples, respectively. As expected, enzymatic activity was higher at higher storage temperature, showing inhibited activity with EO release from active paper sheets. As previously mentioned, competitive inhibition of EOs within the active PPO sites has been demonstrated [11]. Although such studies of the effects of EOs on PPO activity (and other quality-degrading enzymes) are still scarce, the inhibitory effect of clove EO and eugenol has been recently observed in water chestnut and fresh-cut lettuce [11,49]. Nevertheless, our data showed that PPO activity was 20% higher in EO samples compared to CTRL after 26 days at 8 °C. Such behavior may be explained by two hypotheses: (1) the higher enzymatic substrate content (total phenolic content) of EO samples at 8 °C in the previous sampling time led to this enhanced enzymatic activity; (2) more intense tissue deterioration (due to the higher storage temperature or EO-mediated cell damage), leading to changes in membrane permeability and interaction between phenols and PPO (generally found in separate compartments in the cell).

An initial total phenolic content of 126.1 mg kg^−1^ was found (Table 3), which is in the same range as in previous data for other Paraguayo cv. [5]. Storage time and the storage temperature × packaging treatment interaction were significant for total phenolic content (Table 1). Nevertheless, no remarkable total phenolic content changes (<10 mg kg^−1^) were observed in the first 5 days of storage, in accordance with PPO activity. The most remarkable total phenolic content change (53% increment) was observed after commercialization of EO samples previously stored for 5 days at 8 °C. EOs might be understood as an abiotic stress by plant cells, which may respond with an antioxidant reaction, enhancing the biosynthesis of antioxidant compounds, such as phenolic compounds. Carvacrol (the major component of the EO mix used in the inclusion complex) is considered one of the major antioxidant components of EOs [68]. Thus, carvacrol may act as a competitive oxidation protectant of phenolic substrates that can help the use of the accumulated phenolic compounds be avoided due to the abiotic stress response.

The total content of identified major phenolic compounds was 262.6 mg kg^−1^. The lower phenolic content determined by the colorimetric reaction with the Folin–Ciocalteu reagent (126.1 mg kg^−1^) might be owing to the lower reaction of such phenolic compounds with this analytical method, and the quantification with an equivalent single phenolic compound—gallic acid. The individual phenolic compound profiles followed this abundance gradation: catechin > cyanidin-3-glucoside > chlorogenic acid > neochlorogenic acid > cyanidin-3-rutinoside (Table 3). Catechin accounted for 66% of the phenolic total, followed by cyanidin-3-glucoside at 16% of the phenolic total. Neochlorogenic and chlorogenic acids accounted for 7 and 8% of the phenolic total, respectively.

In general, the packaging treatment×storage time interaction was significant for all individual phenolic compounds (except for cyanidin-3-rutinoside). Cyanidin-3-glucoside is an anthocyanin responsible for purple-pink colorations of several horticultural products. As observed in Figure 3, the flesh coloration observed in our samples after commercialization of samples cold-stored for 5 days is in accordance with the increment of cyanidin-3-glucoside (Table 3). In particular, a 206% increment of cyanidin-3-glucoside content was observed after commercialization of EO samples stored for 5 days at 8 °C (compared to values after cold storage for 5 days), as previously discussed for the total phenolic content. The other major anthocyanin (cyanidin-3-rutinoside) also increased greatly (35%) after commercialization of EO samples stored for 5 days at 8 °C. The other phenolic compounds, namely chlorogenic and neochlorogenic acids, also showed similar increments of 128 and 63% in those samples after commercialization, probably as a consequence of the mentioned abiotic stress related to EOs. Finally, the similar PPO activity levels among these samples (prior and after commercialization for 5-day cold storage samples) might discredit the hypothesis of a lower use (leading to the observed higher values) of these phenolic substrates for PPO. Interestingly, high chlorogenic contents were observed in CTRL samples after 5 days at 2 °C, which could be explained as an initial metabolic response to the abiotic stress generated by the low cold storage temperature. Nevertheless, EOs released from the active sheets probably inhibited the phenyl–ammonia lyase activity, the key enzyme in the phenylpropanoid biosynthesis pathway, which has been widely shown to be enhanced by several abiotic stresses, such as low storage temperatures [69].

### 3.7. Total Antioxidant Capacity

An initial total antioxidant capacity of 774 µmol kg^−1^ was found (Table 3), which is in agreement with previous data for flat peaches at the commercial ripening stage [5]. Phenolic compounds are supposed to account for a major portion of the antioxidant activity in many plants. The antioxidant activity of phenolic compounds is related to their highly conjugated structure, but likewise is related to glycosylation and hydroxylation patterns [5]. In accordance, the total phenolic content was highly correlated, with an R value (Pearson’s correlation coefficient) of 0.86 (Table 3). A similar high correlation (R = 0.86–0.96) between the total phenolic content and total antioxidant capacity determined by the DPPH method was previously reported for flat peaches [5,70]. The correlation between the sum of individual phenolic compounds and total antioxidant capacity was slightly reduced (R = 0.69) compared to total phenolic content, as assessed using the Folin–Ciocalteu colorimetric method. This finding highlights the contributions of other antioxidant compounds (e.g., ascorbic acid and some enzymatic systems—superoxide dismutase, catalase, etc.) to the total antioxidant capacity, since those compounds are also able to react during the Folin–Ciocalteu colorimetric method [71]. As expected, the correlations of individual phenolic compounds with the total antioxidant capacity were lower (R = 0.17–0.50), with cyanidin-3-rutinoside and neochlorogenic acid showing the lowest correlations.

## 4. Conclusions

The aim of the use of active EO paper sheets was to maintain the quality of flat peaches during cold storage and the complimentary commercialization simulation (at room temperature). Furthermore, this study firstly shows the beneficial inhibitory effects of EOs on key enzymes and substrates of the biosynthesis pathway of ethylene, the autocatalytic compound responsible for fruit and vegetable ripening and senescence processes during postharvest life. A similar inhibitory effect was observed on the quality-degrading enzymes polygalacturonase and polyphenoloxidase, which were also linked to corresponding textural and appearance or color changes, respectively. Furthermore, EOs even enriched the bioactive profile (mainly phenolic compounds) of flat peaches as an antioxidant response to such volatile compounds, which was probably interpreted by plant cells as an abiotic stress. This study was conducted under controlled pilot plant scale conditions, but is of special interest for its extrapolation to the industrial scale. 

## Figures and Tables

**Table 1:** Analysis of variance of parameters measured during cold storage periods (2 and 8 °C) and complimentary commercialization simulations (4 days at 22 °C) of flat peaches with or without active paper sheets (including βCD-encapsulated EOs). Values represent least significant differences. ACC, 1-aminocyclopropanecarboxylic acid; MACC, N-malonyl-ACC; ACO, ACC oxidase; PG, polygalacturonase; PPO, polyphenoloxidase.

	Single Factors and Interactions						
	Storage Temperature (A)	Package Treatment (B)	Storage Time (C)	A × B	A × C	B × C	A × B × C
Respiration processes							
Ethylene production	ns	2.7 †	5.7 ‡	3.9 †	8.0 ‡	ns	ns
CO_2_ production	7.1 ‡	7.1 ‡	11.3 ‡	ns	15.9 ‡	12.1 †	ns
ACC	0.11 ‡	0.11 ‡	0.14 ‡	ns	0.19 ‡	0.19 ‡	0.27 ‡
MACC	0.11 ‡	0.08 †	0.14 ‡	ns	0.20 ‡	0.14 ‡	0.28 ‡
ACO	0.006 ‡	0.006 ‡	0.008 ‡	0.009 ‡	0.011 ‡	0.011 ‡	0.011 †
Physicochemical quality							
Weight loss	0.3 ‡	0.3 ‡	0.3 ‡	0.4 ‡	0.4 ‡	0.4 ‡	0.6 ‡
Firmness	1.2 ‡	1.2 ‡	1.5 ‡	ns	2.1 ‡	1.2 *	ns
Soluble solids	ns	ns	ns	ns	ns	ns	ns
pH	ns	0.05 †	0.08 ‡	ns	0.09 †	0.12 ‡	ns
Titratable acidity	ns	ns	0.4 ‡	ns	0.5 †	ns	ns
Color index	ns	0.2 ‡	0.3 ‡	ns	ns	0.4 ‡	ns
Enzymes							
Endo-PG	0.09 *	0.09 *	0.20 ‡	ns	0.16 *	ns	ns
Exo-PG	3.0 ‡	3.0 ‡	3.7 ‡	ns	5.3 ‡	5.3 ‡	ns
PPO	0.06 ‡	ns	0.08 ‡	0.09 ‡	0.11 ‡	0.08 †	0.16 ‡
Phenolic contents							
Total phenolic content	7.5 †	ns	6.8 *	ns	9.6 *	9.6 *	ns
Sum individual phenolics	9.1 ‡	6.8 †	11.1 ‡	7.1 *	15.7 ‡	15.7 ‡	16.6 ‡
Cyanidin-3-glucoside	2.7 ‡	2.7 ‡	3.3 ‡	2.9 †	3.5 †	4.7 ‡	6.6 ‡
Cyanidin-3-rutinoside	ns	0.13 *	0.30 ‡	0.34 ‡	ns	0.42 ‡	0.59 ‡
Catechin	ns	ns	ns	ns	11.5 ‡	ns	ns
Neochlorogenic acid	1.51 ‡	0.83 *	1.85 ‡	2.14 ‡	2.62 ‡	2.62 ‡	3.71 ‡
Chlorogenic acid	1.62 ‡	1.62 ‡	1.98 ‡	2.29 ‡	2.80 ‡	2.80 ‡	3.96 ‡
Total antioxidant capacity	45.1 *	ns	55.2 †	ns	57.6 *	ns	147.7 ‡

Note: ns: not significant; *, †, ‡: significance for *p* ≤ 0.05, 0.01, and 0.001, respectively.

**Table 2:** Soluble solids content (SSC; °Brix), titratable acidity (TA; %), pH, color index, weight loss (%), and firmness (N) values during cold storage periods (2 and 8 °C) and complimentary commercialization simulations (COM; 4 days at 22 °C) for flat peaches with or without active paper sheets (including βCD-encapsulated EOs) (*n* = 3 ± SD).

Storage Time	Storage Temperature		Packaging Treatment	SSC	TA	pH	Color Index	Weight Loss	Firmness
Processing day				12.0 ± 0.1	4.2 ± 0.2	4.19 ± 0.03	2.1 ± 0.1	-	25.9 ± 1.7
5 days	2 °C		CTRL	12.2 ± 0.6	4.0 ± 0.2	4.22 ± 0.02	2.7 ± 0.1	0.53 ± 0.09	14.9 ± 1.2
	2 °C		EOs	12.2 ± 0.6	4.0 ± 0.2	4.27 ± 0.02	2.3 ± 0.2	0.26 ± 0.05	17.4 ± 1.7
	8 °C		CTRL	12.7 ± 0.6	3.8 ± 0.3	4.29 ± 0.03	2.7 ± 0.3	0.09 ± 0.13	13.4 ± 0.8
	8 °C		EOs	12.1 ± 0.3	3.5 ± 0.1	4.35 ± 0.02	2.0 ± 0.3	0.04 ± 0.01	16.2 ± 0.4
5 days + COM	2 °C		CTRL	12.2 ± 0.1	3.8 ± 0.1	4.25 ± 0.02	2.4 ± 0.1	2.65 ± 0.18	13.0 ± 1.8
	2 °C		EOs	12.2 ± 0.1	3.8 ± 0.1	4.29 ± 0.01	2.5 ± 0.1	1.10 ± 0.26	13.8 ± 1.8
	8 °C		CTRL	12.5 ± 0.8	3.7 ± 0.1	4.37 ± 0.04	2.2 ± 0.3	2.61 ± 0.04	10.4 ± 1.3
	8 °C		EOs	12.1 ± 0.5	3.1 ± 0.2	4.39 ± 0.02	2.3 ± 0.1	1.04 ± 0.05	12.1 ± 0.7
26 days	2 °C		CTRL	12.3 ± 0.3	1.7 ± 0.1	4.96 ± 0.04	2.7 ± 0.2	3.94 ± 0.14	10.8 ± 1.4
	2 °C		EOs	12.3 ± 0.2	2.0 ± 0.1	4.73 ± 0.02	2.0 ± 0.1	1.28 ± 0.39	12.1 ± 0.9
	8 °C		CTRL	12.7 ± 1.0	2.1 ± 0.1	4.88 ± 0.07	2.9 ± 0.1	5.52 ± 0.16	5.3 ± 1.1
	8 °C		EOs	12.4 ± 0.6	2.6 ± 0.2	4.66 ± 0.14	2.0 ± 0.1	1.04 ± 0.12	6.8 ± 1.1
26 days + COM	2 °C		CTRL	12.6 ± 0.1	2.0 ± 0.1	5.08 ± 0.01	3.1 ± 0.3	6.54 ± 0.29	6.8 ± 0.3
	2 °C		EOs	12.5 ± 0.3	2.2 ± 0.2	4.88 ± 0.05	2.0 ± 0.1	1.86 ± 0.08	8.3 ± 1.1
	8 °C		CTRL	-	-	-	-	-	-
	8 °C		EOs	12.7 ± 1.0	2.4 ± 0.1	4.69 ± 0.02	1.9 ± 0.4	5.17 ± 0.11	-

**Table 3:** Total phenolic content (TPC), cyanidin-3-glucoside (Cy-3-GLC), cyanidin-3-rutinoside (Cy-3-RUT), catechin, neochlorogenic acid (3-CQA), chlorogenic acid (5-CQA), sum of individual phenolic compounds, and total antioxidant capacity (TAC) values during cold storage periods (2 and 8 °C) and corresponding commercialization simulations (COM; 4 days at 22 °C) for flat peaches with or without active paper sheets (including βCD-encapsulated EOs) (*n* = 3 ± SD).

Storage Time	Temperature	Packaging Treatment	TPC	Cy-3-GLC	Cy-3-RUT	Catechin	3-CQA	5-CQA	Sum	TAC
Processing day			126.1 ± 3.8	42.7 ± 1.6	6.4 ± 0.1	174.1 ± 6.9	17.4 ± 0.4	22.1 ± 0.2	262.6 ± 10.2	774.2 ± 20.0
5 days	2 °C	CTRL	126.9 ± 0.7	37.0 ± 1.6	5.3 ± 0.3	162.5 ± 9.9	28.4 ± 1.9	23.9 ± 1.3	257.2 ± 14.8	736.9 ± 43.0
	2 °C	EOs	118.3 ± 12.4	39.8 ± 4.1	6.3 ± 0.1	167.6 ± 1.9	9.8 ± 0.7	9.8 ± 1.3	233.2 ± 8.0	839.2 ± 58.6
	8 °C	CTRL	125.9 ± 12.2	42.2 ± 1.5	6.3 ± 0.2	175.5 ± 3.7	10.3 ± 1.8	20.5 ± 0.5	254.8 ± 7.3	760.3 ± 48.7
	8 °C	EOs	109.6 ± 8.5	22.0 ± 0.8	5.7 ± 0.1	178.3 ± 4.3	12.8 ± 0.7	9.2 ± 1.6	227.9 ± 6.3	607.7 ± 62.5
5 days + COM	2 °C	CTRL	118.1 ± 9.0	57.1 ± 3.3	6.6 ± 0.1	173.9 ± 1.2	5.1 ± 0.8	4.7 ± 0.5	247.4 ± 11.8	572.0 ± 41.9
	2 °C	EOs	116.3 ± 8.2	38.6 ± 1.1	5.8 ± 0.1	179.7 ± 0.8	12.7 ± 0.9	7.7 ± 0.6	244.6 ± 14.2	622.7 ± 24.7
	8 °C	CTRL	139.4 ± 0.3	58.0 ± 3.8	5.5 ± 0.1	176.1 ± 2.0	9.0 ± 1.9	13.2 ± 1.8	261.8 ± 8.1	753.3 ± 77.5
	8 °C	EOs	193.3 ± 14.5	67.4 ± 3.1	7.6 ± 0.4	198.0 ± 7.7	20.8 ± 1.6	20.9 ± 0.4	314.8 ± 4.2	1287.1 ± 91.0
26 days	2 °C	CTRL	128.3 ± 8.9	49.1 ± 1.3	6.8 ± 0.1	178.7 ± 7.7	8.9 ± 1.9	18.6 ± 2.5	262.1 ± 10.5	746.3 ± 89.3
	2 °C	EOs	125.2 ± 10.5	39.5 ± 3.4	6.1 ± 0.3	172.3 ± 8.0	12.2 ± 1.1	18.4 ± 1.9	248.5 ± 7.8	710.7 ± 54.7
	8 °C	CTRL	97.8 ± 5.6	40.4 ± 2.1	6.7 ± 0.2	163.0 ± 1.2	5.3 ± 1.1	4.0 ± 1.3	219.4 ± 13.6	593.7 ± 52.2
	8 °C	EOs	119.2 ± 11.1	39.3 ± 2.3	6.0 ± 0.2	167.6 ± 9.0	11.5 ± 1.8	17.1 ± 1.4	241.6 ± 10.8	760.6 ± 34.1
26 days + COM	2 °C	CTRL	88.3 ± 15.5	28.9 ± 4.1	6.2 ± 0.4	168.0 ± 9.4	2.3 ± 0.4	1.5 ± 0.1	207.0 ± 14.3	450.7 ± 77.7
	2 °C	EOs	105.9 ± 13.6	21.1 ± 2.4	5.5 ± 0.1	165.9 ± 10.0	6.2 ± 0.5	4.3 ± 0.6	203.0 ± 2.6	480.7 ± 16.3
	8 °C	CTRL	-	-	-	-	-	-	-	-
	8 °C	EOs	139.1 ± 4.1	13.9 ± 0.1	5.2 ± 0.1	185.6 ± 5.0	18.4 ± 0.5	16.6 ± 0.4	239.7 ± 10.2	915.2 ± 22.0
**TAC corelations**			0.86	0.47	0.17	0.50	0.37	0.41	0.69	-
